# The effect of the anti-leukemia inhibitory factor on the immune system in the Balb/c mice bearing breast cancer induced with 4T1 cells

**DOI:** 10.1186/s40001-023-01196-2

**Published:** 2023-07-01

**Authors:** Abolfazl Yavari, Fateme Zare, Hossein Hadinedoushan, Mohammad Taher Tahoori

**Affiliations:** 1 Reproductive Immunology Research Center, Shahid Sadoughi University of Medical Science, Daneshjou Boulevard., Yazd, Iran; 2 Department of Immunology, School of Medicine, Shahid Sadoughi University of Medical Sciences, International Campus, Yazd, Iran; 3 Department of Immunology, School of Medicine, Shahid Sadoughi University of Medical Sciences, Yazd, Iran

**Keywords:** Anti-LIF, Breast cancer, Balb/c mouse, Doxorubicin

## Abstract

**Background:**

Breast cancer is one of the most common cancers. Leukemia inhibitory factor (LIF) is considered as one of the effective factors in the growth of breast cancer, and anti-leukemia inhibitory factor antibody is considered as one of the treatment options for this type of cancer.

**Methods:**

Mice models of breast cancer were made with 4T1 cell line and were randomly divided into four groups. The first group included the mice that received anti-LIF (Anti LIF group). The mice in the second group received anti-LIF and doxorubicin (Anti LIF & DOX). The mice in the third group received only doxorubicin (DOX). Finally, the mice in the fourth group did not receive any intervention. 22 days after tumor induction, some of the mice were killed, and their tumor tissues, lymph nodes, and spleens were separated for evaluating *P53, Caspase-3, TIM-3, LAG-3, CTLA-4,* and* PD-1* genes expression. The percentage of regulatory T cells and level of interferon gamma (IFN-γ) and transforming growth factor-beta (TGF-β) were evaluated. The rest of the mice were kept to check the tumor size and their survival rate.

**Results:**

The proposed intervention did not have any significant effect on the tumor growth and the survival rate. However, the expression of *P53* gene and *Caspase-3* in the tumor tissue of the Anti LIF group had a significant enhancement. In tumor tissues and lymph nodes, the expression of *T-bet, PD-1, TIM-3,* and *LAG-3* genes in the Anti LIF group showed a significant increase. There was no significant difference between groups in the percentage of regulatory T cells and level of IFN-γ and TGF-β.

**Conclusions:**

The proposed interventions were able to have a direct effect on tumors, but no significant effect was observed on the immune system.

## Background

Breast cancer is diagnosed as one of the broadest cancers in 154 out of 185 countries. Evidence suggests that breast cancer can be considered the dominant cause of death in over 100 countries [1]. Leukemia inhibitory factor (LIF), as one of the interleukin (IL)-6 family cytokine, is pleiotropic. Its receptor comprises the LIF receptor β and gp130. This receptor is also utilized by other cytokines such as cardiotrophin-like cytokine, oncostatin M, ciliary neurotrophic growth factor, and cardiotrophin1 [2]. Cytokines, through intervention with many signaling pathways, have a substantial role in the development of tumor growth. Indeed, LIF, as a member of IL-6 superfamily, plays a significant role in tumor growth [3]. There were indicated expressions of LIF and LIFRβ in many solid tumors such as nasopharyngeal, skin, colorectal, and breast cancers. In addition, high expression level of LIF is related toh radioresistance, tumor recurrence, LIF-induced radioresistance, and prevention of DNA repair on these cells in vitro [2]. In cases with breast cancer, a number of researches indicate that LIF can promote tumor condition. LIF enhanced the colony formation and proliferation of MCF7 and T47D cells in a dose-dependent manner in vitro, and anti-LIF antibodies can inhibit this effect on cells [4].

Estrogen receptor (ER), progesterone receptor (PR), and human epidermal growth factor receptor 2 (HER2) expression are used to categorize and treat breast cancer. These indicators' existence has facilitated the creation of effective and tailored treatments. Chemotherapy is the only systemic therapeutic option for tumors that lack expression of ER, PR, and overexpression of HER2, collectively known as triple-negative breast cancer (TNBC) [5]. TNBC is more common in younger individuals than the other BC subtypes, has a faster rate of proliferation, and more frequently metastasizes to the brain, liver, and lungs [6, 7].

To study TNBC and develop effective therapeutic strategies, it is crucial to utilize preclinical models that closely mimic the characteristics and behavior of human TNBC. The 4T1 cell line is widely recognized as an appropriate model for TNBC research. These cells were derived from a spontaneous mammary tumor in a Balb/c mouse and exhibit key features observed in human TNBC, including rapid growth, high metastatic potential, and the ability to spontaneously metastasize to distant sites such as lungs, liver, and bone [8, 9].

By utilizing the 4T1 cell line, we aimed to create a mouse model of breast cancer that closely recapitulates the aggressive nature and metastatic behavior of TNBC in humans. This model enables us to investigate the importance of leukemia inhibitory factor's function in the development of breast cancer, development of drug resistance, and its possible effect in regulating the immune system related to the tumor. If regulation of the immune response against the tumor becomes possible by the function of this cytokine through an antibody against it, we can improve the response to treatment. Doxorubicin, used commonly in chemotherapy, with antibody against LIF could be proposed as an adjuvant therapy in cancer.

## Methods

### Animals

Forty-eight female 4- to 6-week-old inbred BALB*/*c mice with a weight of 16–18 g were purchased from the Pasture Institute of Iran (Tehran, Iran). They were randomly divided into four groups (9 mice for each group) for treatment after tumor induction with a specific protocol*.* We kept the mice in plastic cages where they had access to food and water freely with a 12 h light*/*dark cycle throughout the study. The room temperature and humidity were kept at 23 ± 1^ °^C and 55 ± 10%, respectively.

### Cell line and reagents

Mouse mammary tumor (4T1) was also provided from Pasteur Institute (Tehran, Iran). The cells were seeded at 37 °C in a humidified atmosphere with 5% CO2. 4T1 cells were cultured in RPMI 1640 medium (Sigma Aldrich) supplemented with penicillin (100 units), 10% fetal bovine serum (FBS, Gibco company), and 100 μg/ml of streptomycin. The culture medium was changed every 2–3 days until the confluence of 80% of the cells. Then, the cells were used for tumor induction in mice. For diluting anti-LIF, we used sera of hyper-immunized rabbits with LIF, using affinity chromatography (Sigma-Aldrich).

### Tumorigenesis and treatment

All mice were kept for 7 days for environmental adaptation before starting the injections. After adaptation, the animals were inoculated into their left flanks with 4T1 cells (1 × 10^6^ in 100 µL phosphate-buffered saline (PBS) using a 26G hypodermic needle. Regular checks were performed to assess tumor growth until the palpable tumor was detected. Then, the BALB/c mice were injected intraperitoneally with 10 μg of anti-LIF and 7 mg/kg doxorubicin intravenous as experimental groups. The control group of mice (*N* = 9) was treated with PBS. All mice were injected on the 6th, 9th, 12th, 15th, 18th, and 21st day after tumor inclusion. On the 20th day, doxorubicin was injected to the experimental groups. Six mice from each group were killed on the day 22 after tumor inclusion for further assessments and the rest were kept to monitor their survival rate and tumor size, compared with the controls. The tumor size was estimated by the formula: tumor volume = 0.5 (length × width × height).

### Gene expression

To investigate the apoptotic-related and immune-related genes, the tumor and lymph node and spleen tissues were removed from the killed mice in all groups and applied for total RNA extraction using an easy-to-use cDNA Synthesis kit (Parstous biotechnology, Iran) according to its instruction. Also to evaluate the purity and concentration of extracted RNAs, a nanodrop was used in the absorbance of the A260/A280 ratio and 260 nm, respectively (Bio TeK,USA), using Revert Aid First Strand cDNA Synthesis Kit (parstous biotechnology, Iran). All complementary DNA (cDNA) was synthesized from 1 μg RNA. The cDNA product was kept at – 20  C until use. The primer sequences are summarized in Table 1.

**Table 1:** The sequences of primers

Number	Gene	Accession numbers	Primer sequence (5'-3')	PCR product (bp)
1	*CTLA-4*	NM_001281976.1	F: CTCTGAAGCCATACAGGTGA	200
			R:GGTAATCTAGGAAGCCCACTG	
2	*PD-1*	NM_008798.3	F: GTGGCATCTACCTCTGTGG	277
			R: GTGTCGTCCTTGCTTCCAGC	
3	*TIM-3*	NM_134250.2	F: TCTCCAAGAACCCTAACCAC	160
			R: CAGAGACTCCCACTCCAATG	
4	*LAG-3*	NM_008479.2	F: CTCAATGCCACTGTCACG	180
			R: CTCCTGAATCTCCAGCACAG	
5	*GAPDH*	NM_001289726.1	F: CACTGCCACCCAGAAGACTG	147
			R: CCAGTGAGCTTCCCGTTCAG	
6	*T-bet*	NM_019507.2	F: TCAACCAGCACCAGACAGA	109
			R: AACATCCTGTAATGGCTTGT	
7	*P53*	NM_011640.3	F: GTATTTCACCCTCAAGATCC	84
			R: TGGGCATCCTTTAACTCTA	
8	*Caspase-3*	NM_001284409.1	F: CTCGCTCTGGTACGGATGTG	201
			R:TCCCATAAATGACCCCTTCAT	

To determine the mRNA levels of *Caspase-3, P53*, *T-bet, CTLA-4, PD-1, TIM-3,* and *LAG-3* in both case and control groups, a quantitative real-time polymerase chain reaction (qRT-PCR) method was carried out. *GAPDH* was considered as a reference gene for the normalization of six target genes expression. Master Mix Green with high ROX™ (Amplicon) was utilized for PCR reaction, using the StepOne system (Applied Biosystems, CA, USA). Each PCR run was performed in a final volume of 20 µL containing cDNA (2 μL), forward primer (1 µL), reverse primer (1 µL), master mix (10 µL), and 6 µL nuclease-free water. All run methods consisted of one cycle of holding stage (10 min at 95 °C), followed by 35 cycles of amplification stage at 95 °C for 15 s, 60 °C for 30 s, and 72 °C for 30 s. A melting curve stage was run after the cycling stage in the range of 60–95 °C to verify the specificity of the amplicons. The relative expression level of each gene was analyzed by the 2^−△△Ct^ method.

### Cytokine assays

Six Balb/c mice in each group were killed on day 22 after tumor incubation. Each blood sample were collected in a tube and centrifuged. The resultant serum was stored at − 20 °C until assay. The levels of interferon gamma (IFN-γ) and transforming growth factor-beta (TGF-β) were determined by sandwich enzyme-linked immunosorbent assay (ELISA). For the assay, the following combinations of capture and biotinylated mAbs were used as recommended by the manufacturer (KPG-MIFN, KPG-HMTG-ß, Iran). The amount of cytokines was calculated using standard murine recombinant cytokine curves run on the the same immunoplate.

### Preparation of single-cell suspension and flow cytometry assays

After euthanizing three mice per group, their spleens were removed. By smoothly homogenizing spleens with the top of a 5-mL syringe as a plunger, we could collect single cells using a cell strainer under sterile conditions. Red blood cells from the cell suspension were removed by adding distilled water and 10X PBS (9 mL × 1 mL). After that, we centrifuged the cell suspension at 500 g for 5 min at room temperature, and splenocytes in the cell pellets were re-suspended in RPMI-1640 medium supplemented with 10% FBS. We stained splenocytes with anti-mouse CD4-PrecP, anti-mouse CD25-APC, anti-mouse FoxP3-PE (Biolegend, USA). After 15 min fixation and incubation, we used Perm/Wash for washing. We use intracellular anti bodies for 30 min in 4 °C with Perm/Wash. At the end, we used BD FACS Calibur (BD biosciences, USA) for evaluating stained splenocytes.

### Statistical analysis

Statistical analyses were carried out with IBM SPSS 22 Statistics (IBM SPSS, NY, USA). All values were shown as mean ± SEM. The normal distribution was assessed by Kolmogorov–Smirnov and Shapiro–Wilk tests. Statistical significance was assessed by Kruskal–Wallis and also Tukey HSD was employed. A log-rank (Mantel–Cox) test was used to compare the survival rate of the mouse. *P* < 0.05 was considered a significant value.

## Results

**Fig. 1:**
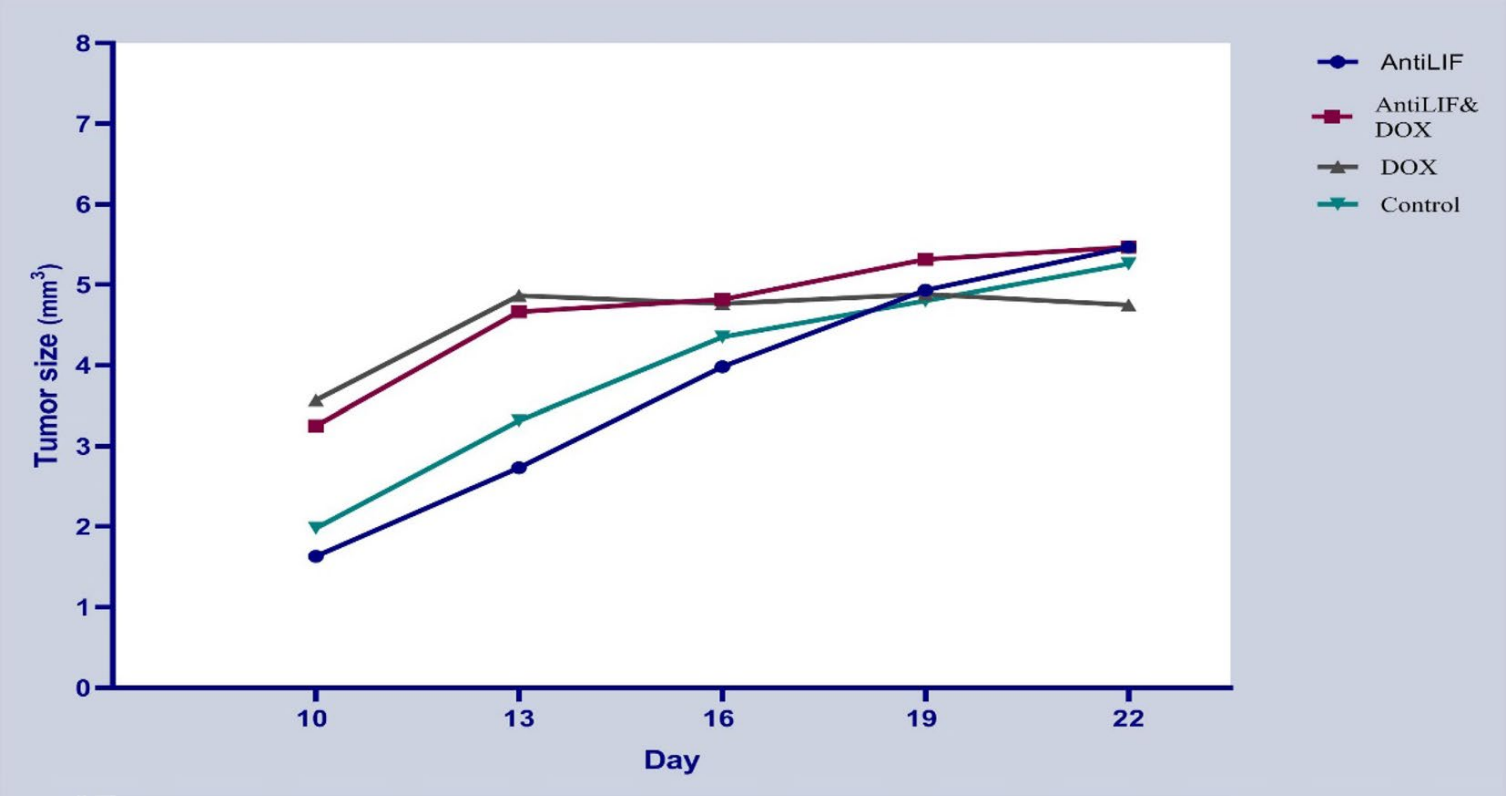
Protective effects of anti-LIF and doxorubicin on tumor growth. *Anti LIF* mice treated with anti-LIF, *Anti LIF & DOX* mice treated with anti-LIF and doxorubicin_,_ *DOX* mice treated with doxorubicin, control: mice treated with phosphate buffer saline. The data were presented as mean ± SEM

**Fig. 2:**
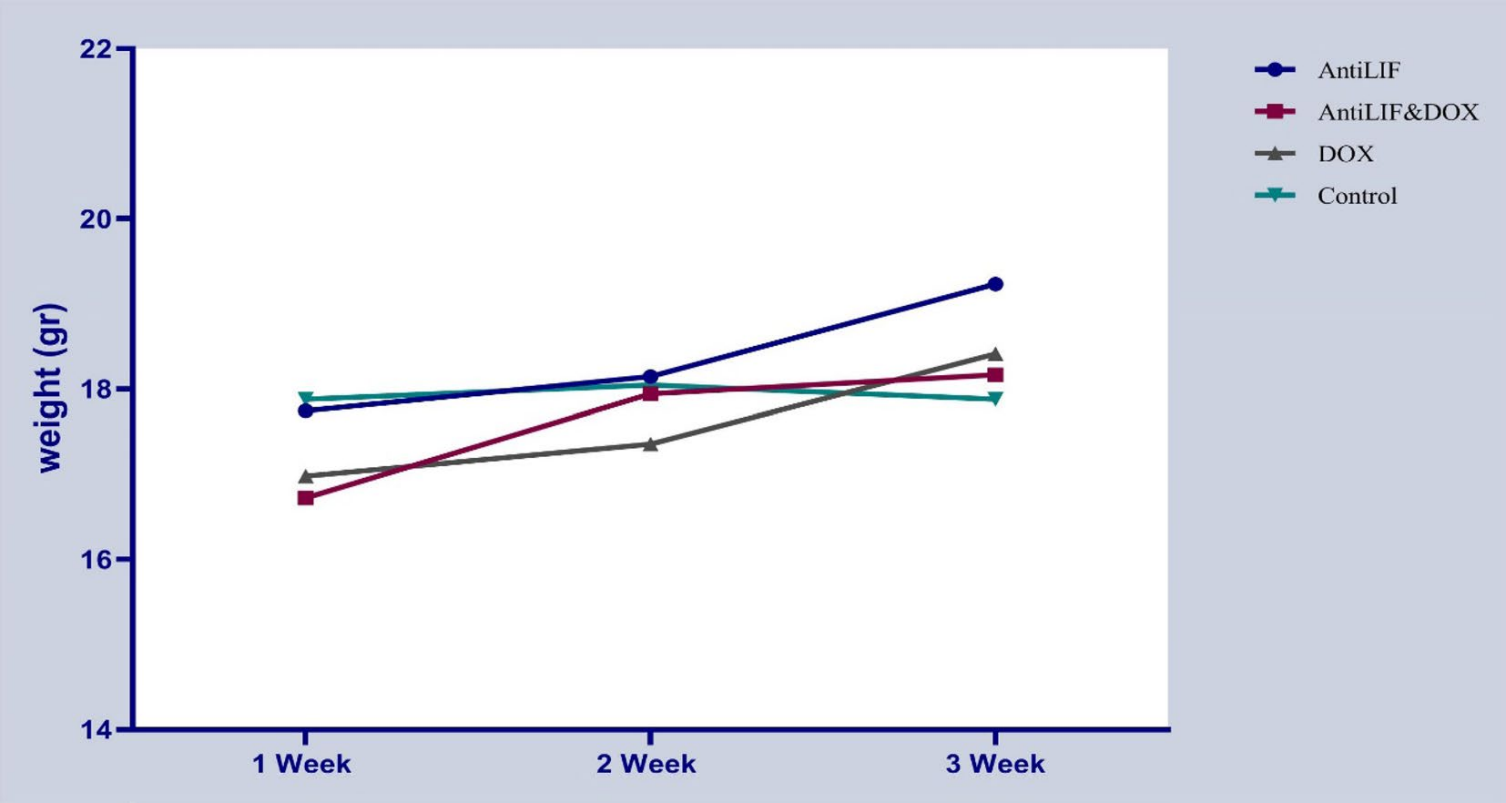
Protective effects of anti-LIF and doxorubicin on weight of mice. *Anti LIF* mice treated with anti-LIF, *Anti LIF & DOX* mice treated with anti-LIF and doxorubicin_,_ DOX: mice treated with doxorubicin, control: mice treated with phosphate buffer saline. The data were presented as mean ± SEM

Experimental groups and control group did not have any significant difference in the tumor growth and weight of mice (Figs. 1, 2). The survival rates of mice were not significantly prolonged in the experimental groups in comparison to the controls. There was no significant difference among the experimental groups concerning the survival rates of the mice (Table 2).

**Table 2:** Protective effects of different doses of anti-LIF and doxorubicin on the survival rates of mice in each study group

Groups	Mean				Median			
	Estimate	Mean SD	95% confidence interval		Estimate	Mean SD	95% Confidence Interval	
			Lower limit	Upper limit			Lower limit	Upper limit
Anti LIF	70.66	16.33	38.65	102.68	63	13.06	37.39	88.6
Anti LIF & DOX	50.66	1.2	48.31	53.02	50	0.81	48.4	51.6
DOX	51.66	7.83	36.3	67.03	59	18.77	22.19	95.8
Control	60.33	7.53	45.56	75.1	56	4.89	46.39	65.6

*Anti LIF & DOX* mice treated with anti-LIF and doxorubicin_,_ *DOX* mice treated with doxorubicin, control: mice treated with phosphate buffer saline. The data were presented as mean ± SEM

**Fig. 3:**
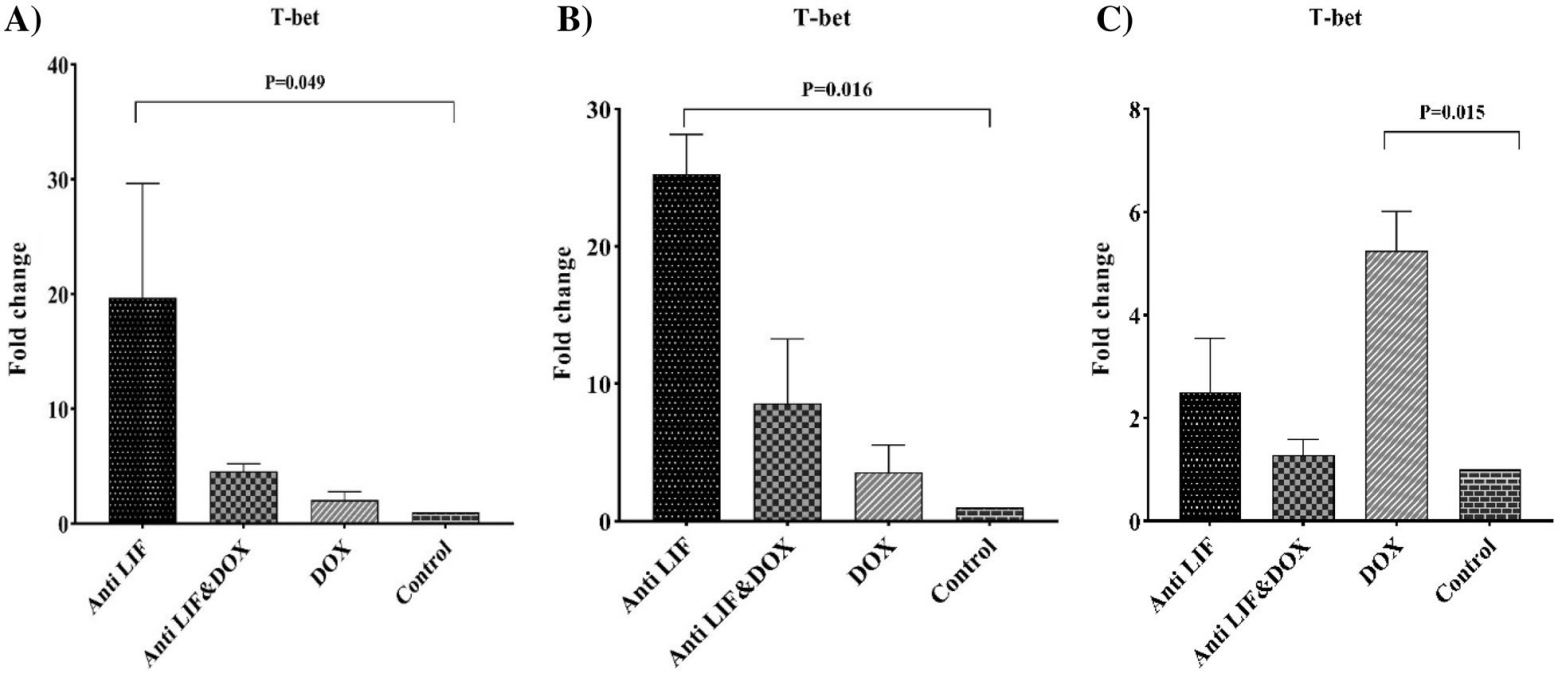
**A** mRNA level of *T-bet* in the tumor tissues, **B** mRNA level of *T-bet* in the lymph node tissues, **C** mRNA level of *T-bet* in the spleen tissues. *Anti LIF* mice treated with anti-LIF, *Anti LIF & DOX* mice treated with anti-LIF and doxorubicin_,_ *DOX* mice treated with doxorubicin, control: mice treated with phosphate buffer saline. The data were presented as mean ± SEM

Considering the expression of *T-bet* in lymph node and tumor tissues, it was found that the highest level of expression of this gene belonged to the Anti LIF group and similarly the expression of this gene was significantly more than that of the control group. In spleens, the DOX group had higher expression in comparison with the control group (Fig. 3).

**Fig. 4:**
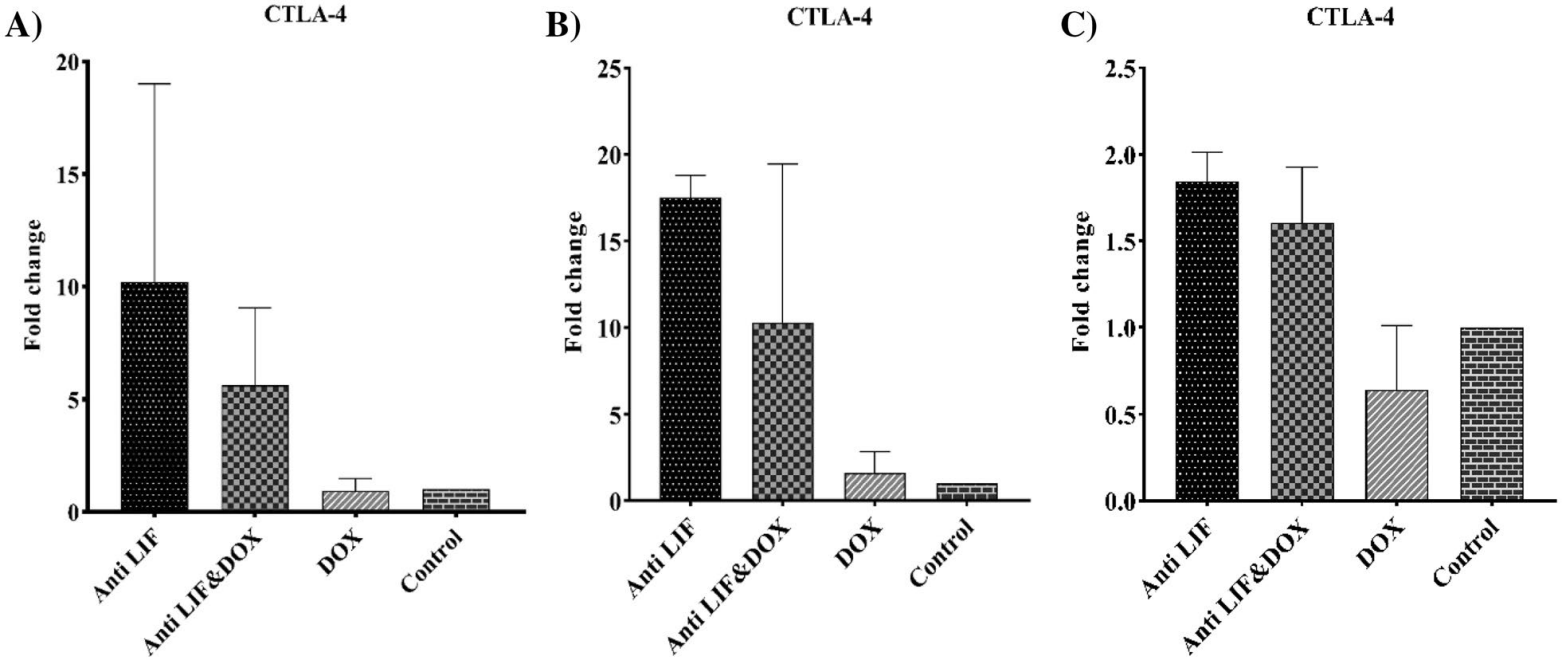
**A** mRNA level of *CTLA-4* in the tumor tissues, **B** mRNA level of *CTLA-4* in the lymph node tissues, **C** mRNA level of *CTLA-4* in the spleen tissues. *Anti LIF* mice treated with anti-LIF, *Anti LIF & DOX* mice treated with anti-LIF and doxorubicin_,_ *DOX* mice treated with doxorubicin, control: mice treated with phosphate buffer saline. The data were presented as mean ± SEM

Regarding the expression of *CTLA-4*, the mRNA level of this gene was not significant in all groups. The highest level of expression of this gene was seen in the Anti LIF group (Fig. 4).

**Fig. 5:**
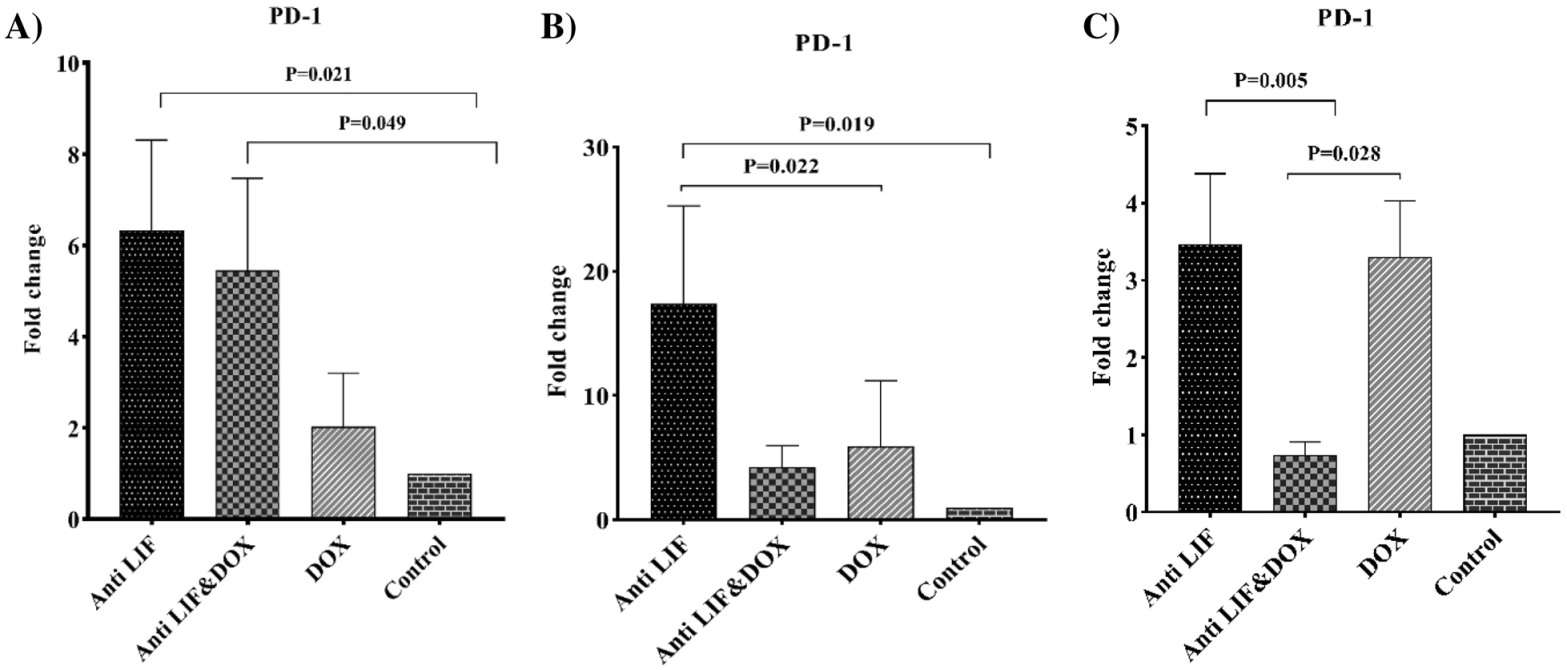
**A** mRNA level of *PD-1* in the tumor tissues, **B** mRNA level of *PD-1* in the lymph node tissues, **C** mRNA level of *PD-1* in the spleen tissues. Anti LIF: mice treated with anti-LIF, Anti LIF & DOX_:_ mice treated with anti-LIF and doxorubicin_,_ DOX: mice treated with doxorubicin, Control: mice treated with phosphate buffer saline. The data were presented as mean ± SEM

Considering the *PD-1* mRNA level, in both tissues, RNA level of PD*-1* was increased in the Anti LIF in comparison with the control group. While in tumor tissue, the RNA level of *PD-1* was enhanced in the Anti LIF & DOX compared to the control group, in lymph nodes, doxorubicin in DOX group can significantly decrease the expression level of *PD-1* in comparison with the Anti LIF group. In spleens, Anti LIF & DOX showed significantly lower expression compared to the DOX group and Anti LIF group (Fig. 5).

**Fig. 6:**
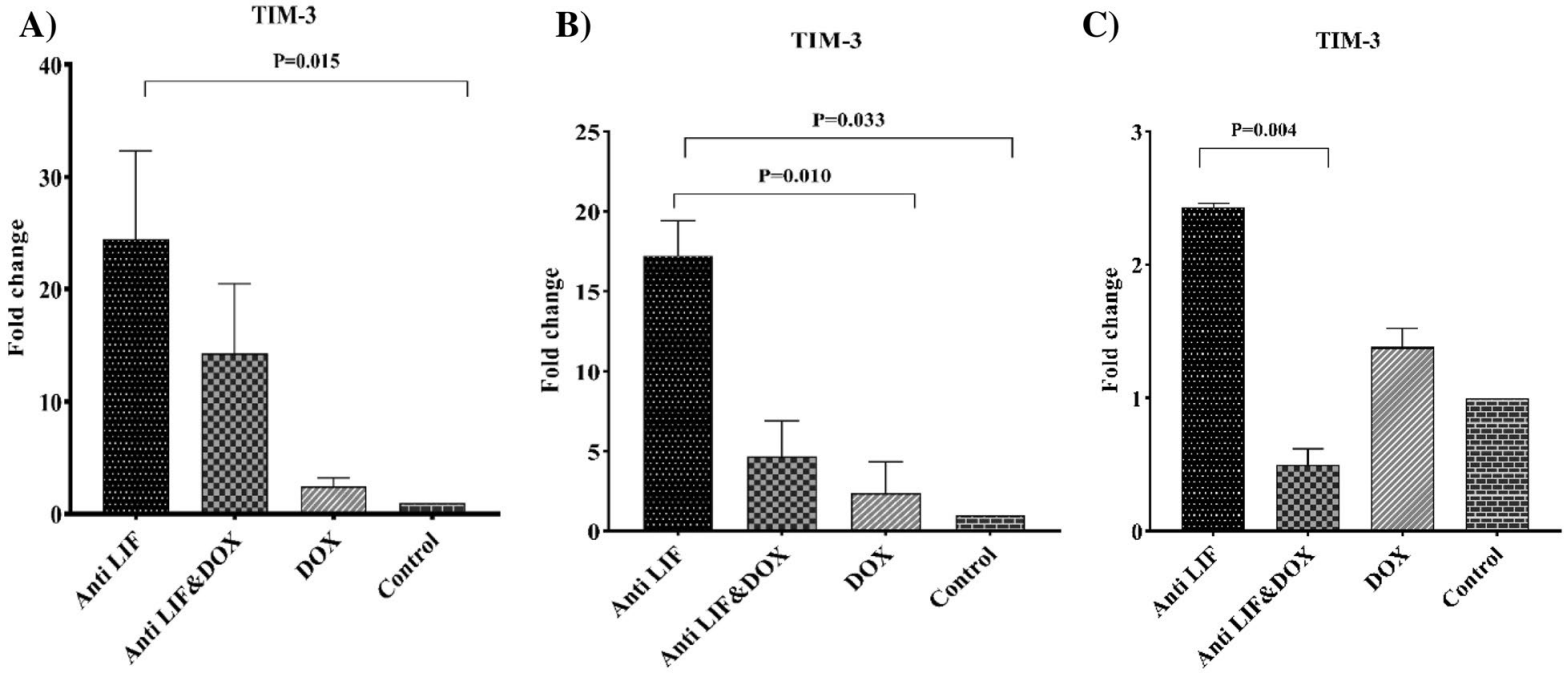
**A** mRNA level of *TIM-3* in the tumor tissues, **B** mRNA level of *TIM-3* in the lymph node tissues, **C** mRNA level of *TIM-3* in the spleen tissues. *Anti LIF* mice treated with anti-LIF, *Anti LIF & DOX* mice treated with anti-LIF and doxorubicin_,_ *DOX* mice treated with doxorubicin, control: mice treated with phosphate buffer saline. The data were presented as mean ± SEM

Regarding the *TIM-3* expression level, in both tissues, *TIM-3* mRNA level was significantly increased in the Anti LIF group compared to the control group. In the lymph nodes, mRNA level of this gene was significantly lower than that of the Anti LIF group. In spleens, the expression of this gene in the Anti LIF & DOX group was lower than that of the Anti LIF group (Fig. 6).

**Fig. 7:**
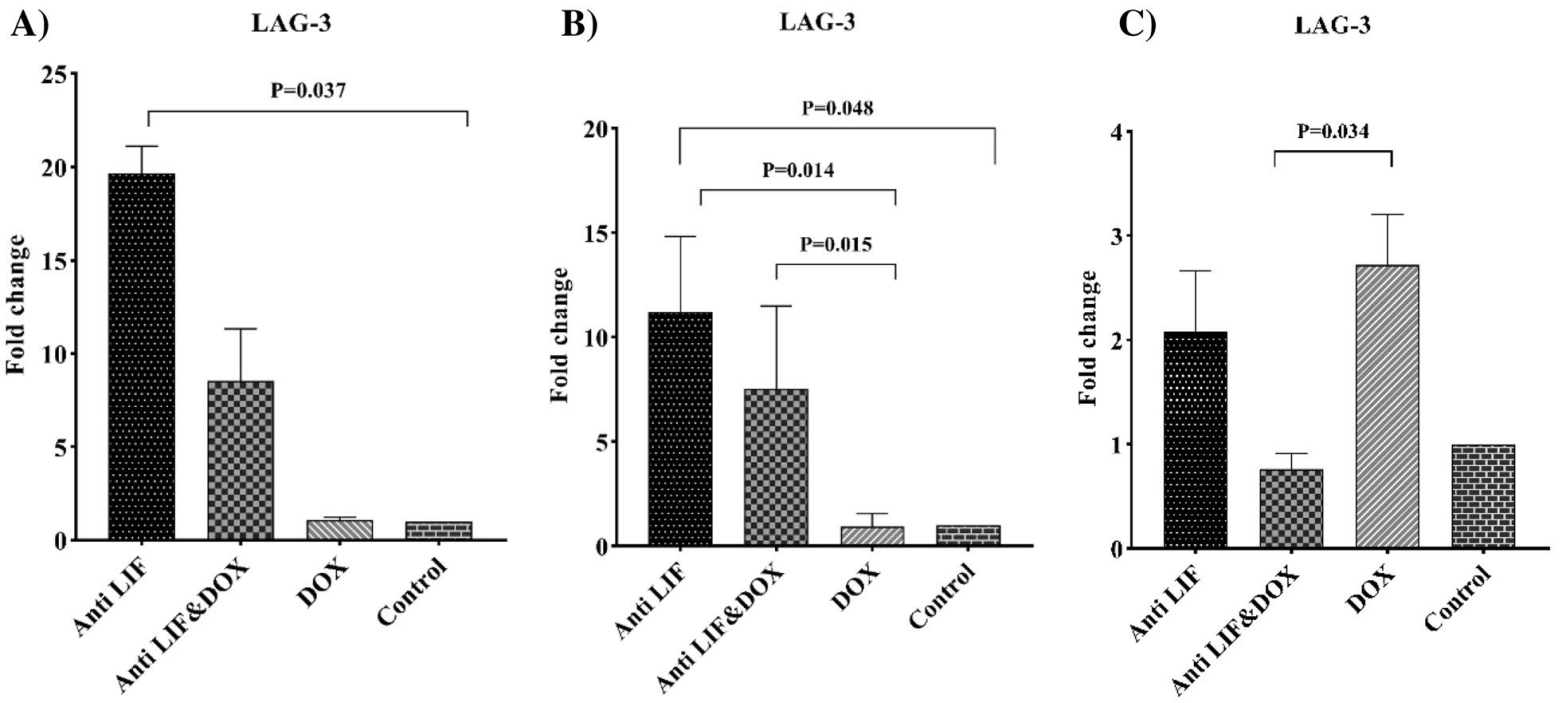
**A** mRNA level of *LAG-3* in the tumor tissues, **B** mRNA level of *LAG-3* in the lymph node tissues, **C** mRNA level of *LAG-3* in the spleen tissues. *Anti LIF* mice treated with anti-LIF, *Anti LIF & DOX* mice treated with anti-LIF and doxorubicin_,_ *DOX* mice treated with doxorubicin, control: mice treated with phosphate buffer saline. The data were presented as mean ± SEM

With respect to the *LAG-3* mRNA level, in lymph nodes, the expression level of *LAG-3* was significantly lower in the DOX group compared to both the Anti LIF & DOX and Anti LIF groups. In both tissues, mRNA level of *LAG-3* was significantly increased in the Anti LIF group. In spleens, Anti LIF & DOX showed significantly lower expression compared to the DOX group (Fig. 7).

**Fig. 8:**
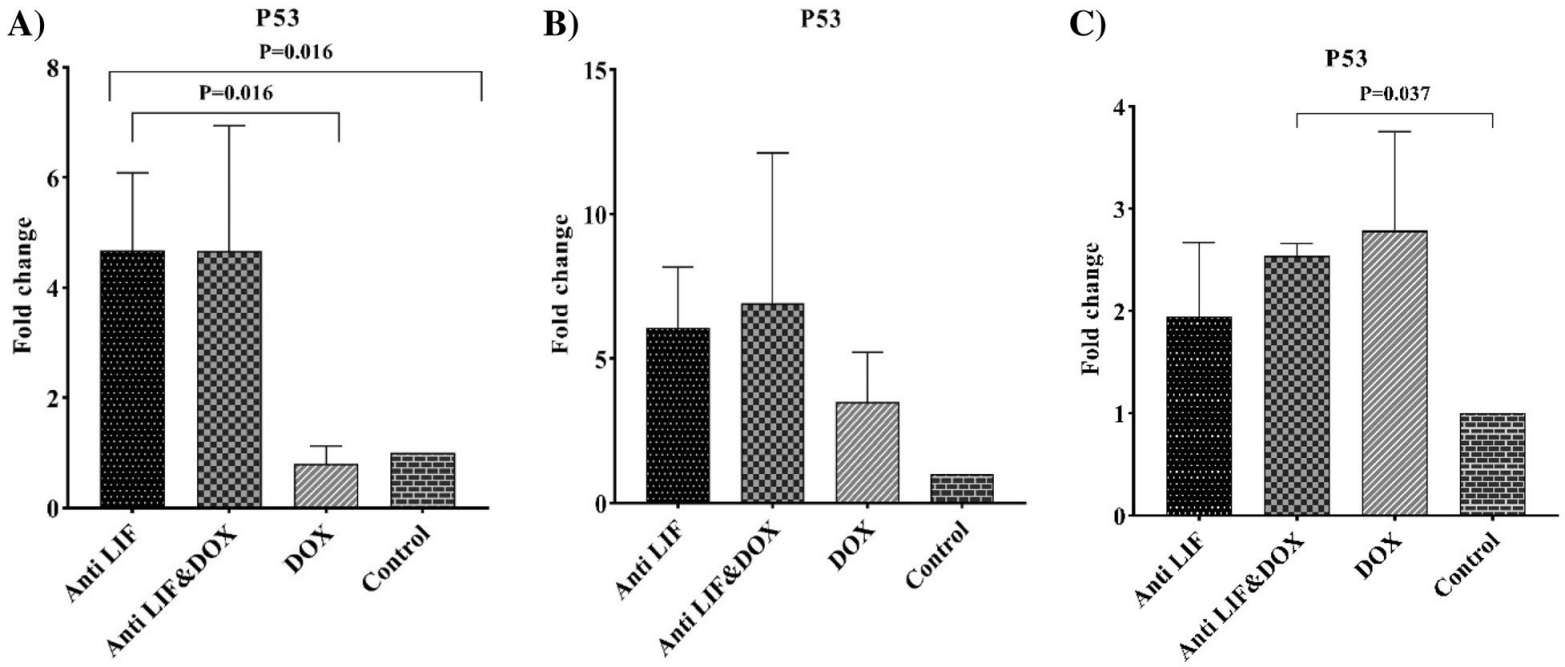
A) mRNA level of *P53* in the tumor tissues, **B** mRNA level of *P53* in the lymph node tissues, C) mRNA level of *P53* in the spleen tissues. Anti LIF: mice treated with anti- LIF, Anti LIF & DOX_:_ mice treated with anti-LIF and doxorubicin_,_ DOX: mice treated with doxorubicin, control: mice treated with phosphate buffer saline. The data were presented as mean ± SEM

**Fig. 9:**
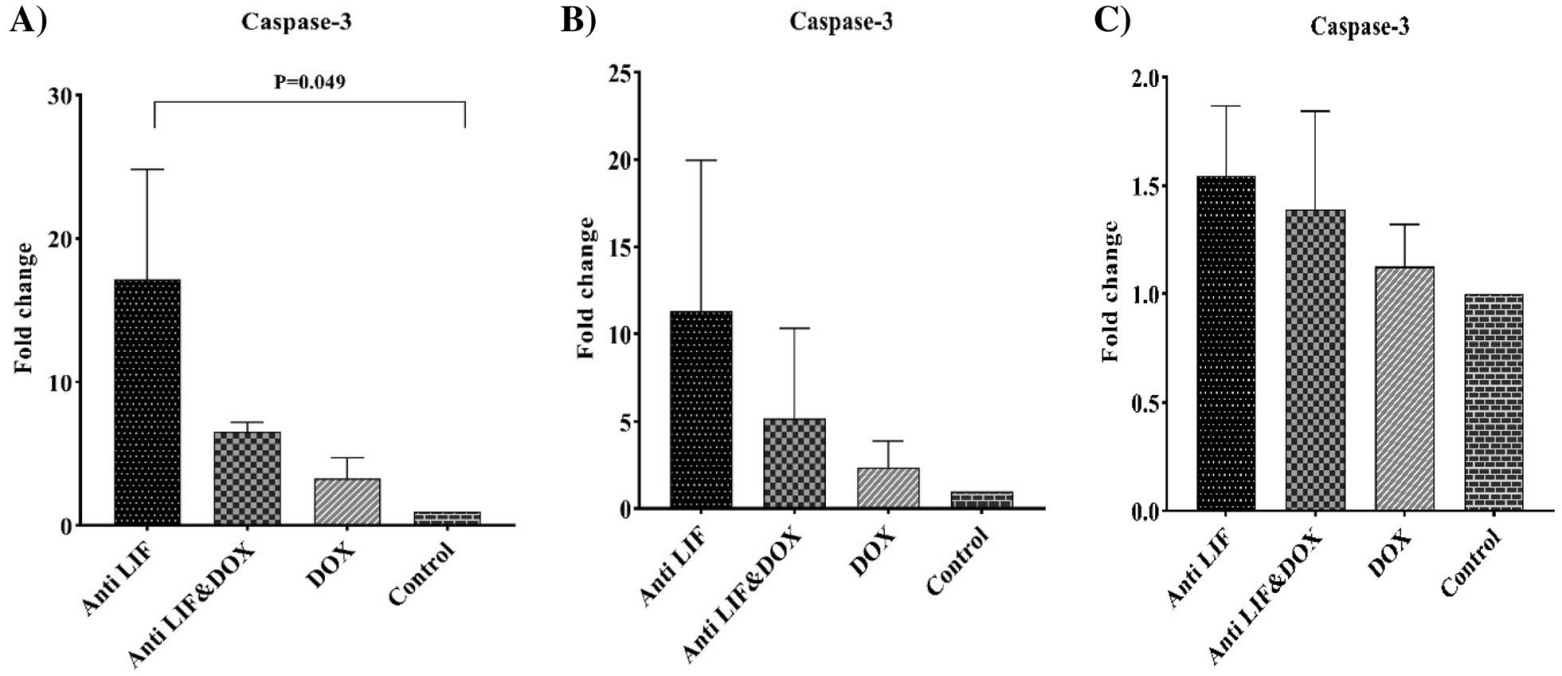
**A** mRNA level of *Caspase-3* in the tumor tissues, **B** mRNA level of *Caspase-3* in the lymph node tissues, **C** mRNA level of *Caspase-3* in the spleen tissues. *Anti LIF* mice treated with anti-LIF, *Anti LIF & DOX* mice treated with anti-LIF and doxorubicin_,_ *DOX* mice treated with doxorubicin, control: mice treated with phosphate buffer saline. The data were presented as mean ± SEM

In tumor tissues, the highest mRNA levels of *P53* belonged to the Anti LIF group compared to the control group, whereas the expression of this gene was significantly lower in the DOX group compared to the Anti LIF. In spleens, the Anti LIF & DOX group showed significantly higher expression level compared to the control group (Fig. 8). In lymph nodes and spleens, no significant difference was observed among the groups. In tumor tissues, the expression of mRNA of *Caspase 3* was significantly increased in the Anti LIF group compared to the control group, while in the lymph nodes, no significant mRNA level change was seen among the groups (Fig. 9).

**Fig. 10:**
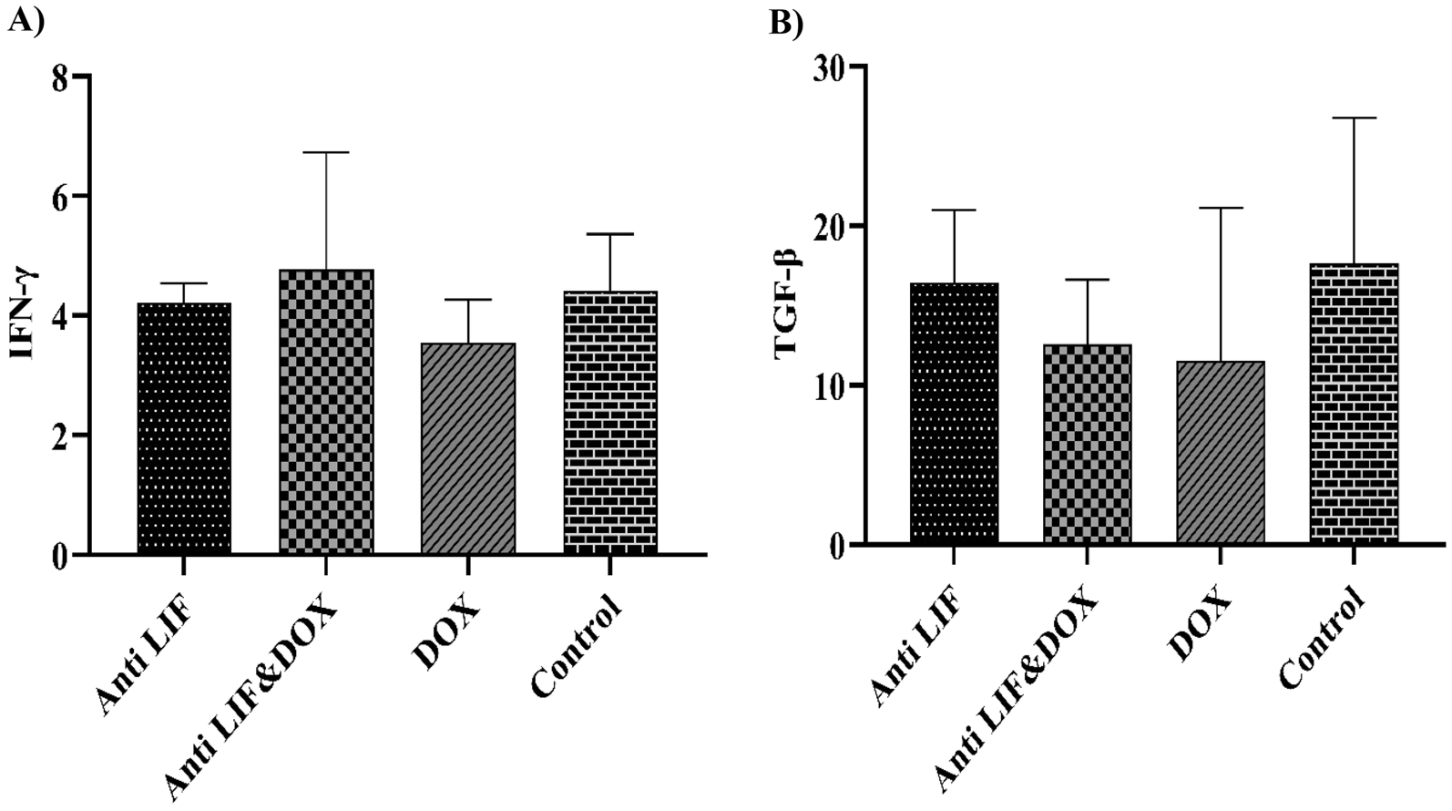
**A** Serum level of IFN-γ, **B** serum level of TGF-β. *Anti LIF* mice treated with anti-LIF, Anti LIF & Doxorubicin: mice treated with anti-LIF and doxorubicin_,_ Doxorubicin: mice treated with doxorubicin, Control: mice treated with phosphate buffer saline. The data were presented as Mean ± SEM

The levels of cytokines in the sera were compared between the different groups. The mean value for IFN-γ in the control group was 4.41 ± 0.94 Pg/ml (mean ± SEM). The mice in the treated groups did not show any significant difference. The highest serum level of IFN-γ was shown in the group which received both anti-LIF and doxorubicin (4.77 ± 1.95). Similarly, the mean value for TGF-β in the control group was 17.63 ± 9.14 Pg/ml (mean ± SEM). There was no significant difference between the groups. The lowest level of this cytokine was seen in the group which received doxorubicin (11.54 ± 9.58). The results are shown in Fig. 10.

**Fig. 11:**
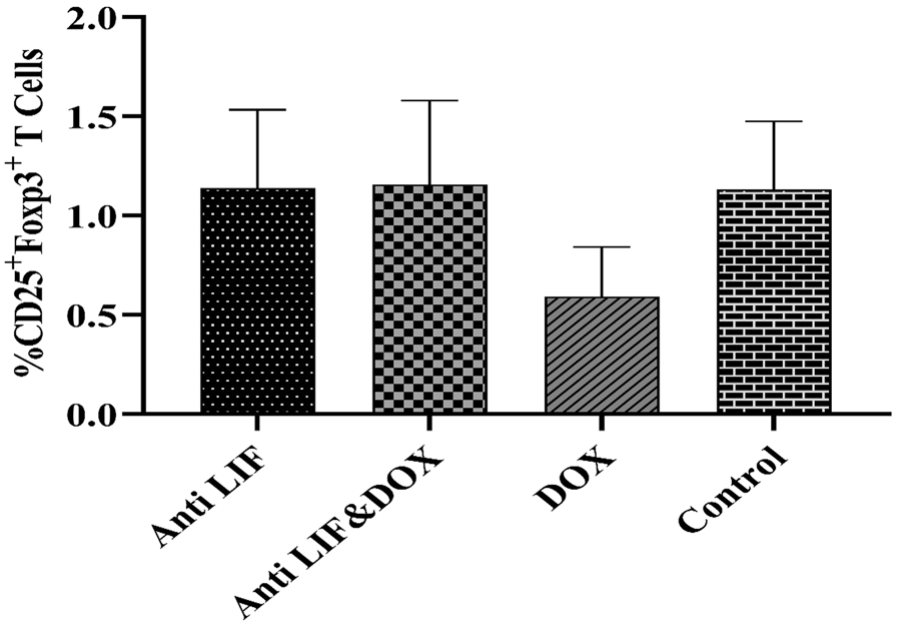
Percentage of regulatory T cells. Anti LIF: mice treated with anti-LIF, Anti LIF & Doxorubicin_:_ mice treated with anti-LIF and doxorubicin_,_ Doxorubicin: mice treated with doxorubicin, control: mice treated with phosphate buffer saline. The data were presented as mean ± SEM

Regarding the assessments of T regulatory cells using flow cytometry technique, our results showed no significant change in the level of T regulatory cells between different groups. Also, the data showed the highest percentage of T regulatory cells in the group which received both anti-LIF and doxorubicin (1.15 ± 0.42). Conversely, the lowest percentage of T regulatory cells was shown in the group which only received doxorubicin (0.59 ± 0.24) (Fig. 11).

## Discussion

plenty number of studies have shown the overexpression of LIF at the protein levels and mRNA in human breast cancer [10, 11]. Progestins and antiprogestin can regulate the LIF level and the proliferation of some estrogen-dependent (MCF-7 and T47D) and estrogen-independent (SK-BR3 and BT20) breast cancer cell lines and fresh breast carcinoma cells can be affected by the LIF level [12, 13]. In a related study, the expression of LIF and LIFR in 50 human breast cancer specimens was assessed. With immunohistochemical techniques, the expression level of LIF and LIFR in tumors was found to be 78% and 80%. LIF and LIFR are highly expressed in breast tumors, in comparison with normal specimen and, consequently, this kind of expression has some relationships desirable biological characteristics of breast cancers [10]. There are different breast cancer cell lines (HS578T, MCF7, SK-Br-3, MDA-MB-232, MDA–MB-468, BT474, T47D) in which mRNA levels of LIF have been assessed [14]. According to the metastatic ability of different cell lines, the expression level of LIF fluctuates. In cell lines with higher metastatic ability (HS578T and MDA–MB-231), LIF levels are higher compared to less metastatic ones [15]. In another study, it was proved that LIFR level of protein in non-metastatic human breast cancer cell lines (MCF7, SUM159, SUM149, SUM229) was higher than in lower ones (SUM1315 and MDA–MB-231) [16]. In the present study, it has been tried to find out the effect of the Anti LIF on the immune system in the Balb/c mice bearing breast cancer induced 4T1 cells.

In terms of evaluating the beneficial effects of anti-LIF and doxorubicin on tumor growth as well as survival rates of mice with breast cancer, the data of the current research did not indicate any significant decrease in tumor size in different groups compared to the controls. However, Wang et al. showed that the simultaneous use of anti-LIF and a usual chemotherapy drug, gemcitabine, can significantly increase the life span of mice model of pancreatic cancer and significantly decrease the tumor growth of this mice model [17]. This controversy can possibly be attributed to the type of cancer mouse model and the different role of LIF in pancreatic and breast cancer. Moreover, the different mechanism of action of the chemotherapy drug used can also have an effect.

*PD-1*, *TIM-3*, *LAG-3,* and *CTLA-4* are essential molecules in the maintenance of self-tolerance and the regulation of immune responses for decreasing tissue damage [18]. Tumors essentially hijack the immune checkpoint pathway to survive and spread [19]. A number of tumors, especially breast cancers, can express co-inhibitory receptors that are essential for mediating the escape from T cell-mediated immune surveillance [18]. Results of the current study demonstrate that anti-LIF cannot significantly reduce the expression of these co-inhibitory receptors. In contrast to anti-LIF, doxorubicin can effectively reduce mRNA level of these co-inhibitory receptors. Sadeghi et al. examined the role of immune checkpoint inhibitors in the effectiveness of neoadjuvant chemotherapy in the mice model of breast cancer. They administrated different dosages of doxorubicin, carboplatin and paclitaxel, and then assessed the mRNA level of *TIM-3*, *PD-1,* and *CTLA-4* with real-time PCR. In line with our study, doxorubicin can significantly reduce the expression level of *PD-1* and *TIM-3,* but cannot significantly affect *CTLA-4* expression [20]. Li et al. showed that injection of LIF into a mouse model of breast cancer causes more growth and invasion of cancer cells through the AKT–mTOR signaling pathway [14]. In another study, researchers showed LIF can differentiate CD4^+^ naive T cells to regulatory T cells [21]. The immune system has some substantial role in the tumor growth [22]. As a result, one effective way to assess the impact of different interactions is evaluating the expression of these genes. According to our study, anti-LIF cannot significantly reduce tumor growth; hence, LIF promotes tumor growth and T regulatory expansion.

In addition, *T-bet*, which is encoded by the *TBX21* gene, is an immune cell-specific transcription factor that is a member of the T-box transcription factor family. There are different immune cells such as dendritic cells, natural killer (NK) cells, CD4^+^, TCD8^+^ T cells, B lymphocytes, and a subgroup of regulatory T cells [23]. Stimulation of T cell receptors and IL-12 can increase *T-bet* and lead to the regulation of activated T cells. Activated T cells as anti-tumor lymphocytes lead to increased production of cytokines such as IFN γ [24]. Changes in *T-bet* gene expression in tumor tissues and lymph nodes shows that *T-bet* gene expression in Anti LIF group is significantly higher than that in the control group. Also, no significant difference was shown among other groups. In this study, due to the absence of the effect of the treatment on the tumor size and also the survival rate of the mice, it seems that the expression of this gene by miRNAs, especially miRNA-29, has not turned into a functional protein [25].

*TP53* is the most prevalent mutated tumor suppressor gene, whose mutation can initiate tumorigenesis [26]. It is guessed that approximately one-half of human cancers contain a mutation in *P53* [27]. In the present study, the effect of the proposed interaction on the mRNA expression level of *P53* in the tissues obtained from tumors and lymph nodes was found. It was found that the expression level of *P53* gene in the tumor tissues of the studied mice in the Anti LIF group had a significant increase in comparison with the control and doxorubicin groups, but there was no significant difference in the expression of *P53* in tissues obtained from the lymph. Yu et al. showed that in the mice model of colorectal cancer, LIF can significantly decrease the mRNA level of *P53* [28]. In accordance with this study, our results showed that anti-LIF can significantly augment *P53*.

*Caspase-3* is a member of the cysteine protease family, which has an important role in apoptosis. We chose *Caspase-3* expression in breast cancer cells for this research because of its substantial role in apoptosis [29]. In tumor tissues, the level of *Caspase-3* gene expression in the Anti LIF group was significantly higher than that in the control group. However, no significant difference was observed in the expression of genes among different groups in the lymph nodes. In a related study, due to the presence of LIFR and OSMR in the breast cancer cells, LIF and OSM were found to be able to activate STAT3, which converts the phenotype of these cancer cells to malignant phenotype [30]. These impacts can upregulate the Bcl-Xl and suppress apoptosis [31]. The data of this study surprisingly showed that anti-LIF can increase apoptosis with enhancement of *Caspase-3*.

A dimerized soluble cytokine called IFN-γ is the only interferon of the type II class [32]. T helper cells, particularly Th1 cells, cytotoxic T cells, macrophages, mucosal epithelial cells, and NK cells all release IFN-γ. This cytokine is a crucial paracrine signal in the early innate immune response and a crucial autocrine signal for professional antigen presenting cells in the adaptive immune response. The cytokines IL-12, IL-15, IL-18, and type I IFN all work together to increase the expression of IFN-γ [33]. The single type II interferon is IFN-γ, which differs from type I interferons serologically by being acid-labile as opposed to type I variations which is acid stable. IFN-γ possesses antiviral, immune-suppressive, and anti-tumor activities [34]. TGF-β is a versatile cytokine that is a member of the transforming growth factor superfamily, which also comprises three other mammalian isoforms (TGFB1, TGFB2, and TGFB3) and numerous other signaling proteins [35]. TGF-β inhibits the cell cycle at the G1 stage in healthy cells to halt proliferation, induce differentiation, or encourage death. TGF-β no longer regulates the cell in many cancer cells due to mutations in the TGF-β signaling system. These cancerous cells multiply, and additionally the stromal cells (fibroblasts) in the vicinity multiply. These cells increase the amount of TGF-β they produce. The stromal cells, immunological cells, endothelial cells, and smooth muscle cells nearby are all affected by this TGF. It results in immunosuppression and angiogenesis, which increase the invasiveness of the tumor [36]. There was no significant difference between groups in the level of IFN-γ in sera. The Anti LIF & Doxorubicin group had the highest level of this cytokine in comparison with other groups. Regarding the TGF-β level, there was no significant difference between groups. Also, the lowest level of this cytokine was seen in the doxorubicin group.

A specialized subgroup of T cells called regulatory T cells function to inhibit immunological response, preserving homeostasis and self-tolerance. Regulatory T cells have been demonstrated to have the ability to suppress T cell growth and cytokine production, and they are essential for preventing autoimmunity. These cells come in a variety of subgroups with have various functions [37]. In our study, there was no significant difference between groups. Also, the doxorubicin group had the least number of T regulatory cells in comparison with other groups.

## Conclusions

In general, the effect of the drug takes place in two ways: direct effect on the tumor and indirect effect on the immune system. Results of this study show direct effect of the proposed drugs. It is indicated that this drug can increase the expression of the *P53* gene, tumor suppressive protein, and *Caspase-3*, one of the key protease in the apoptotic pathway. With respect to the role of our drugs on the immune system, immune suppressive genes and genes that have a role in the exhaustion of T lymphocytes, such as *PD-1*, *TIM-3,* and *LAG-3*, increased. There is no significant difference between the groups in the percentage of regulatory T cells and level of IFN-γ and TGF-β.

Finally, it can be concluded that anti-LIF along with the drug doxorubicin had a significant effect on some genes studied in this research, but did not have a significant effect on the reduction of tumor size and weight of tumor-bearing mice.

## Data Availability

The data that support the findings of this study are available from the corresponding author upon reasonable request.
